# The Spermine Oxidase/Spermine Axis Coordinates ATG5‐Mediated Autophagy to Orchestrate Renal Senescence and Fibrosis

**DOI:** 10.1002/advs.202306912

**Published:** 2024-05-22

**Authors:** Dan Luo, Xiaohui Lu, Hongyu Li, Yi Li, Yating Wang, Simin Jiang, Guanglan Li, Yiping Xu, Kefei Wu, Xianrui Dou, Qinghua Liu, Wei Chen, Yi Zhou, Haiping Mao

**Affiliations:** ^1^ Department of Nephrology The First Affiliated Hospital Sun Yat‐sen University NHC Key Laboratory of Clinical Nephrology Guangdong Provincial Key Laboratory of Nephrology Guangzhou Guangdong 510080 China; ^2^ Department of Nephrology Shunde Hospital Southern Medical University (The First People's Hospital of Shunde) Foshan Guangdong 528308 China

**Keywords:** autophagy, cell senescence, renal fibrosis, SMOX, spermine

## Abstract

Decreased plasma spermine levels are associated with kidney dysfunction. However, the role of spermine in kidney disease remains largely unknown. Herein, it is demonstrated that spermine oxidase (SMOX), a key enzyme governing polyamine metabolism, is predominantly induced in tubular epithelium of human and mouse fibrotic kidneys, alongside a reduction in renal spermine content in mice. Moreover, renal SMOX expression is positively correlated with kidney fibrosis and function decline in patients with chronic kidney disease. Importantly, supplementation with exogenous spermine or genetically deficient SMOX markedly improves autophagy, reduces senescence, and attenuates fibrosis in mouse kidneys. Further, downregulation of ATG5, a critical component of autophagy, in tubular epithelial cells enhances SMOX expression and reduces spermine in TGF‐β1‐induced fibrogenesis in vitro and kidney fibrosis in vivo. Mechanically, ATG5 readily interacts with SMOX under physiological conditions and in TGF‐β1‐induced fibrogenic responses to preserve cellular spermine levels. Collectively, the findings suggest SMOX/spermine axis is a potential novel therapy to antagonize renal fibrosis, possibly by coordinating autophagy and suppressing senescence.

## Graphical abstract

The relationship between polyamine metabolism and kidney fibrogenesis is elucidated through the spermine oxidase (SMOX)/spermine axis. Supplementation with spermine or genetic deficiency in SMOX improves autophagy, suppresses cell senescence, and alleviates renal fibrosis. Moreover, the interplay between autophagy protein 5 (ATG5) and SMOX plays a crucial role in regulating the progression of renal fibrosis by preserving intracellular spermine levels. Created with BioRender.com.

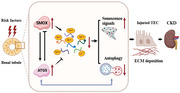

## 1 Introduction

The prevalence of chronic kidney disease (CKD) is gradually increasing worldwide, and CKD may become the fifth leading cause of death in the world by 2040.^[^ ^1^, ^2^ ^]^ Despite the different etiologies, renal fibrosis, characterized by tubular atrophy, myofibroblast activation, and extracellular matrix (ECM) deposition, is the final manifestation of CKD.^[^ ^3^ ^]^ The decline in renal function accompanied by fibrotic pathological changes could be induced by many factors, among which metabolic disorders have emerged as one of the main drivers in recent years.^[^ ^4^, ^5^, ^6^ ^]^ Renal tubular epithelial cells (TECs) are the major targets of various insults, and tubular injury is involved in the initiation and evolution of renal fibrosis.^[^ ^7^ ^]^ Therefore, modulating critical cellular metabolic pathways may provide a novel therapeutic strategy to delay the progression of renal fibrosis.

Unlike metabolites that serve as building blocks or energy sources, polyamine has been suggested to modulate many fundamental cellular processes.^[^ ^8^ ^]^ Spermine, an end‐product of polyamine metabolism, is ubiquitous and its levels are tightly regulated through de novo biosynthesis, catabolism, and transportation across the plasma membrane. Spermine oxidase (SMOX) is a key and highly inducible enzyme in polyamine catabolic pathway. It specifically oxidizes spermine to generate spermidine with toxic byproducts, including hydrogen peroxide and 3‐aminopropanal. Under cell stress conditions, SMOX is upregulated and links to increased reactive oxygen species that can cause cell injury and death.^[^ ^9^, ^10^, ^11^, ^12^ ^]^ Alternatively, spermine is degraded via the spermidine/spermine N1‐acetyltransferase (SAT1), a rate‐limiting enzyme for polyamine reverse transformation.^[^ ^13^ ^]^ Spermine is an organic cation containing multiple amino groups that has a variety of biological functions, including immune regulation, maintaining mitochondrial homeostasis, enhancing autophagy, and promoting cell proliferation and differentiation.^[^ ^14^ ^]^ It has been demonstrated that abnormal spermine metabolism is closely related to the occurrence and development of various human diseases.^[^ ^15^ ^]^ Clinical observations have indicated that the plasma spermine level is significantly decreased in CKD patients and negatively correlated with serum creatinine and urea nitrogen.^[^ ^16^, ^17^ ^]^ Consistently, SMOX activity in the blood is markedly increased in CKD patients.^[^ ^18^ ^]^ Spermine supplementation or targeting its metabolic enzymes could be potential therapeutic strategies for diseases.^[^ ^19^, ^20^, ^21^ ^]^ However, spermine metabolism in the kidney, especially in TECs, which may be involved in the pathogenesis of CKD, has yet to be fully investigated.

In this study, we demonstrated that SMOX expression was upregulated in tubular cells and spermine contents were decreased in fibrotic kidneys of CKD patients and mouse models. Supplementation with exogenous spermine or genetically deficient SMOX improved autophagy, inhibited tubular cell senescence, and ultimately alleviated renal fibrosis. Further, ATG5 negatively regulated SMOX protein expression, and the ATG5/SMOX interaction preserved cellular spermine content. Collectively, our study provides insights into targeting spermine metabolism as a novel promising strategy for retarding the progression of CKD.

## 2 Results

### 2.1 SMOX Is Induced in Injured Kidneys and Is Associated with Fibrogenesis

**Figure 1:**
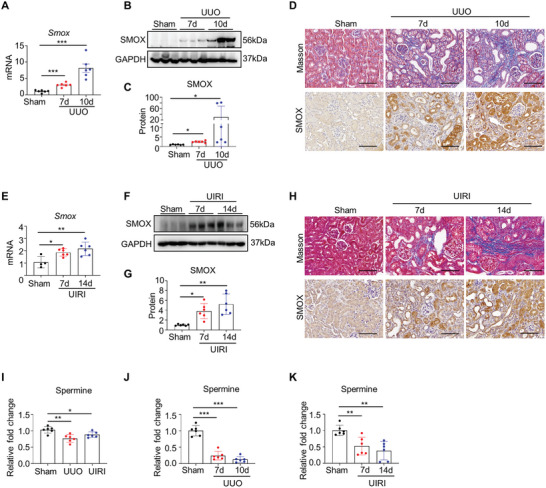
SMOX is induced in renal tubular epithelium in CKD mouse models. A) qRT‐PCR showed the *Smox* mRNA levels in the injured kidney of UUO mice. B,C) Renal SMOX expression was determined by Western blotting. GAPDH was used as a control (*n* = 6). Dot plots represent quantitative densitometric data from Western blotting. D) Representative images of Masson trichrome staining (upper panel) and immunostained for SMOX (lower panel) in kidneys after UUO. Scale bar, 100 µm. E) qRT‐PCR showed the *Smox* mRNA level in the injured kidney of UIRI mice. F,G) Renal SMOX expression was induced at indicated time points after UIRI. GAPDH was used as a control (n = 6). Dot plots represent quantitative densitometric data from western blotting. H) Representative images of Masson trichrome staining (upper panel) and immunostained for SMOX (lower panel) in kidneys after UIRI. Scale bar, 100 µm. I) The relative fold change in spermine in the kidneys of sham surgery, UUO at day 10, or UIRI at day 14 by mass spectrometric detection (*n* = 6). J,K) The relative fold change in spermine in the kidneys at indicated time points after UUO and UIRI by ELISA (*n* = 6). Data are expressed as means ± SEM, *P < 0.05, **P < 0.01, ***P < 0.001 versus sham kidneys. UUO, unilateral ureteral obstruction; UIRI, unilateral renal ischemia‐reperfusion.

Despite reports have shown the tight connection of serum spermine with CKD patients, the SMOX expression and content of spermine in kidneys and their relationship to renal fibrosis have never been directly tested. We employed two renal fibrosis mouse models, the unilateral ureteral obstruction (UUO) and the unilateral renal ischemia‐reperfusion (UIRI), to determine spermine metabolism in the development of kidney fibrosis. The results showed that the mRNA levels of *Smox* were upregulated at day 7 and continued to elevate at day 10 after UUO (**Figure** **1**A). Western blotting analysis confirmed that the protein levels of SMOX were also enhanced in fibrotic kidneys (Figure 1B,C). Immunohistochemical analysis revealed that during fibrosis progression, upregulated SMOX was predominantly concentrated in TECs (Figure 1D). This distribution was further validated in co‐immunostaining for SMOX and the proximal tubular marker LTL (Figure S1A, Supporting Information). Likewise, the expression of SMOX in the kidneys of UIRI mice also gradually increased with progressive fibrosis, showing a similar pattern (Figure 1E–H and Figure S1B, Supporting Information). Next, mass spectrometry and ELISA were performed to accurately determine the changes of spermine content in kidney tissues. The mass spectrometry results showed that the spermine content in fibrotic kidneys of UIRI and UUO mice was decreased compared with that in the normal control (Figure 1I). Moreover, ELISA data revealed a gradual and significant decrease of spermine content in kidneys with prolongated time after UUO and UIRI (Figure 1J,K). These results together demonstrate a disturbance of spermine oxidase/spermine axis in fibrotic kidneys.

**Figure 2:**
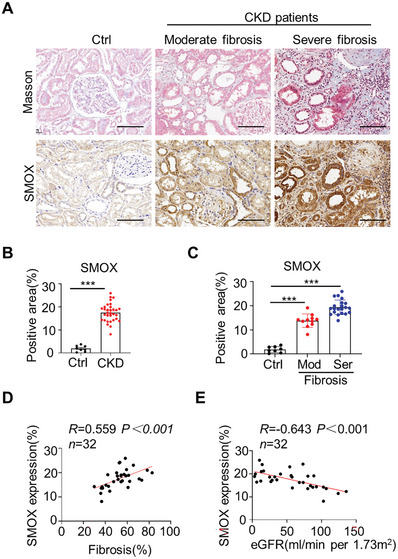
SMOX is elevated in the kidneys of CKD patients. A) Representative images of Masson trichrome staining (upper panel) and immunostained for SMOX (lower panel) in kidney tissues from CKD patients. Non‐tumor kidney tissues from patients with renal carcinoma were used as controls (Ctrl). Scale bar, 100 µm. B,C) Graphical representation showing the relative abundance of SMOX expression in the kidneys of Ctrl and CKD patients with varying degrees of kidney fibrosis. D,E) The correlations between the positive area for D) SMOX staining and renal fibrosis and E) eGFR in CKD patients. The *P* value, number of patients (*n*), and Spearman correlation coefficient (*R*) are indicated on the graph. Data are expressed as means ± SEM, ***P < 0.001 versus the Ctrl group. CKD, chronic kidney disease; eGFR, estimated glomerular filtration rate; Ctrl, control; Mod, moderate; Ser, severe.

To explore the relevance of the primary polyamine catabolic enzymes in human CKD, we performed immunohistochemical staining on renal biopsies from 32 CKD patients and nephrectomized normal tissues. As shown in **Figure** **2**A, SMOX was weakly expressed in normal human kidneys, while it displayed a pronounced expression in renal tubular epithelium of CKD patients, and relatively faint staining was detectable in interstitial cells. Importantly, SMOX expression was significantly increased in kidneys with the aggravation of renal fibrosis (Figure 2B,C). Correlation analysis revealed that SMOX expression was positively correlated with renal fibrosis, and negatively correlated with estimated glomerular filtration rate (eGFR) (Figure 2D,E). In contrast, the protein expression of SAT1, an enzyme that positively regulates spermine catabolism, also appeared to slightly increased but lacked correlation with renal fibrosis and eGFR (Figure S2A,B, Supporting Information). In addition, by mining the published database, renal mRNA levels of *SMOX* and *SAT1* in CKD patients were upregulated (Figure S2C, Supporting Information), suggesting that these enzymes‐mediated abnormal spermine metabolism may be involved in the progression of human CKD.

### 2.2 Exogenous Spermine or Knockdown of SMOX Alleviates Renal Fibrosis in Mice

**Figure 3:**
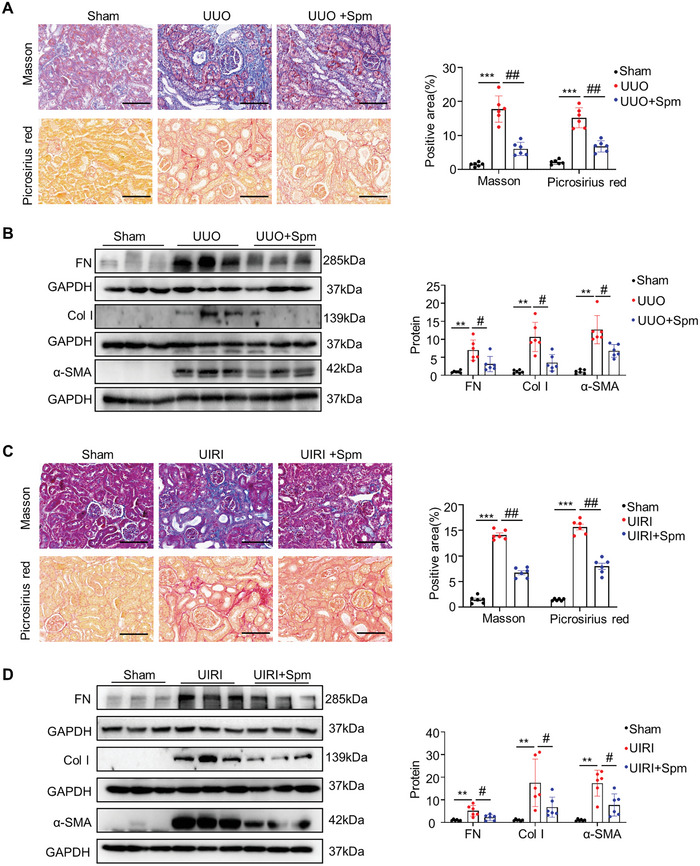
Exogenous spermine alleviates renal fibrosis in CKD mouse models. A) Masson trichrome and picrosirius red staining images in various groups as indicated were shown. Scale bar, 100 µm. Quantitative determination of collagen deposition area based on Masson trichrome and picrosirius red staining in different groups (right panel) (*n* = 6). B) Representative western blotting and quantitative data for fibrosis‐related protein levels of FN, Col I, and α‐SMA in the kidneys of various groups as indicated were shown. C) Masson trichrome and Picrosirius red staining images in various groups as indicated were shown. Scale bar, 100 µm. Quantitative determination of collagen deposition area based on masson trichrome and picrosirius red staining in different groups. D) Representative western blotting and quantitative data for fibrosis‐related protein levels of FN, Col I, and α‐SMA in the kidneys of different groups as indicated are shown. Data are expressed as means ± SEM, **P < 0.01, ***P < 0.001 versus sham controls; #*P* < 0.05, ##*P* < 0.01 versus UUO/UIRI with vehicle (*n* = 6). FN, fibronectin; Col I, collagen I; α‐SMA, α‐smooth muscle actin.

To investigate the effect of spermine supplementation on renal fibrosis, UUO mice were intraperitoneally injected with vehicle or spermine (1 mg kg^−1^) starting 2 d before surgery, and continuously injected daily for 10 d (Figure S3A, Supporting Information). Masson trichrome and Picrosirius red staining revealed substantial collagen deposition in the tubulointerstitium 10 days after UUO compared with sham‐operated mice, which was attenuated by administration of spermine (**Figure** **3**A). Consistently, western blotting analysis showed that UUO‐induced renal expression of fibronectin (FN), collagen I (Col I), and α‐smooth muscle actin (α‐SMA) were dramatically mitigated by spermine (Figure 3B). Similar results were also obtained in UIRI mouse model (Figure 3C,D and Figure S3B, Supporting Information). Further, to assess the therapeutic effect of spermine on renal fibrosis, mice were treated with spermine starting on day 3 after UUO surgery (Figure S3C, Supporting Information). Administration of spermine reduced the extent of fibrosis, as determined by Masson trichrome and Picrosirius red staining, as well as suppressed expression of fibrosis‐related proteins (FN and vimentin) (Figure S3D,E, Supporting Information).

**Figure 4:**
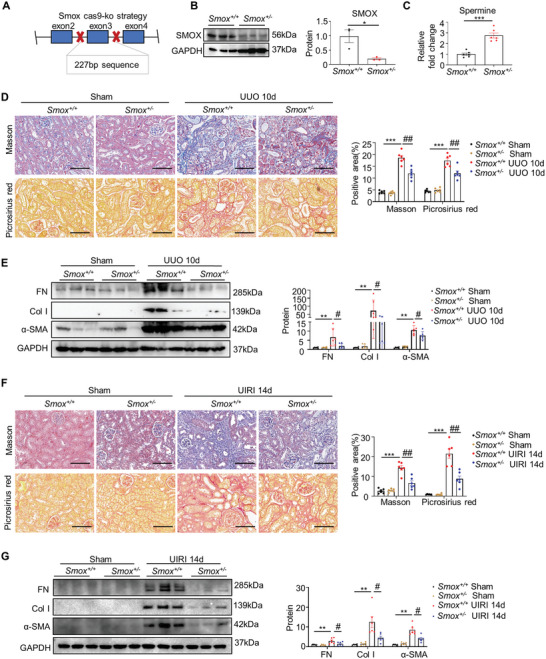
Knockdown of SMOX ameliorates renal fibrosis in UUO and UIRI mice. A) Schematic diagram of the *Smox^+/−^* mouse construction strategy. B) Representative western blotting and quantitative data showing the expression of SMOX in the kidneys of *Smox^+/+^* and *Smox^+/−^* mice. *P < 0.05 versus *Smox^+/+^* (*n* = 3). C) Graphical representations showing the relative fold‐change of spermine in the kidney of *Smox^+/+^* and *Smox^+/−^* mice. D) Representative images of kidney tissues from indicated groups with either Masson trichrome (upper panel) or Picrosirius red staining (lower panel). Scale bar, 100 µm. Quantitative assessment of the collagen deposition area based on masson trichrome and picrosirius red staining in different groups. E) Representative western blotting and quantitative data showing fibrosis‐related protein levels of FN, Col I, and α‐SMA in the kidneys of different groups as indicated are shown. F) Representative images of kidney tissues from indicated groups with either masson trichrome staining (upper panel) or picrosirius red staining (lower panel). Scale bar, 100 µm. Quantitative assessment of the collagen deposition area based on masson trichrome and Picrosirius red staining in different groups. G) Representative western blotting and quantitative data for fibrosis‐related protein levels of FN, Col I, and α‐SMA in the kidneys of different groups as indicated are shown. Data are expressed as means ± SEM, **P < 0.01, ***P < 0.001 versus *Smox^+/+^* sham; #*P* < 0.05, ##*P* < 0.01 versus *Smox^+/+^* mice with either UUO or UIRI (*n* = 6).

Given that increased SMOX expression and decreased spermine level in fibrotic kidneys, as well as the anti‐fibrotic impact of spermine in mouse models of renal fibrosis, we hypothesized that elevated endogenous renal spermine content exerts favorable effects on fibrogenesis. To this end, *Smox* heterozygote (*Smox^+/−^*) mice were constructed by the CRISPR/Cas9 technique (**Figure** **4**A). Western blotting confirmed a significantly decreased SMOX expression in kidneys of *Smox^+/−^* mice, as compared with their wild‐type (*Smox^+/+^*) littermates (Figure 4B). As expected, a substantial increase in spermine concentration measured by ELISA was observed in kidneys from *Smox^+/−^* mice (Figure 4C). HE staining, Masson trichrome, and Picrosirius red staining revealed that there is no obvious difference in kidney morphology between sham‐operated *Smox^+/+^* and *Smox^+/−^* mice (Figure 4D and Figure S4A, Supporting Information). However, SMOX knockdown markedly attenuated UUO‐induced collagen deposition (Figure 4D). Western blotting further verified that expression of profibrotic markers, including FN, Col I, and α‐SMA, were also significantly reduced in the UUO kidneys of *Smox^+/−^* mice (Figure 4E). Similar results were obtained in UIRI mouse model, in which interstitial fibrosis were significantly reduced in the injured kidneys of *Smox^+/−^* UIRI mice in contrast to *Smox^+/+^* mice (Figure 4F,G). These in vivo findings suggest that SMOX/spermine axis is critical for inhibiting renal fibrosis.

### 2.3 Spermine Supplementation or SMOX Depletion Abrogates TGF‐β1‐Induced ECM Production in TECs

**Figure 5:**
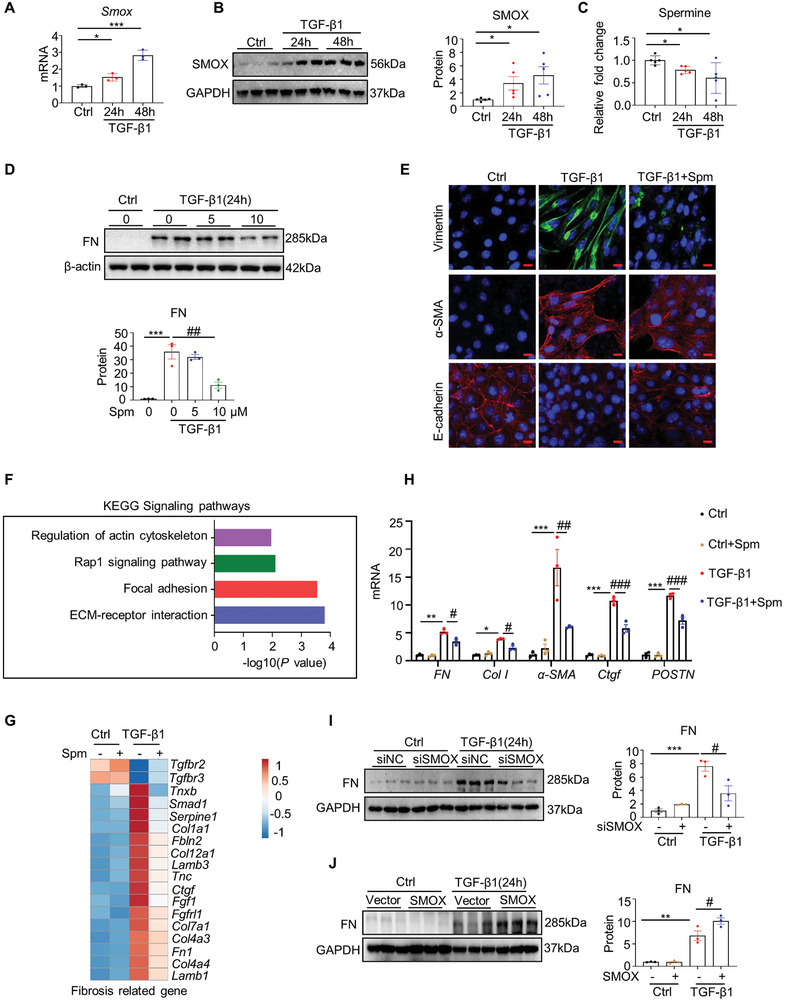
Spermine inhibits TGF‐β1‐induced ECM production in renal tubular cells (mTECs). A,B) Cells were treated with 10 ng/mL of TGF‐β1 for the indicated time periods. The *Smox* mRNA and SMOX protein levels were analyzed by A) qRT‐PCR and B) Western blotting, respectively. *P < 0.05, ***P < 0.001 versus Ctrl (*n* = 3). C) Graphical representation showing the relative level of spermine in mTECs with or without TGF‐β1 stimulation, measured by ELISA. *P < 0.05 versus Ctrl (*n* = 5). D) Cells were preincubated with 5 × 10^−6^ and 10 × 10^−6^ m of spermine (Spm) for 12 h and then treated with 10 ng mL^−1^ of TGF‐β1 for 24 h. Western blotting and quantitative data of FN expression are presented. ***P < 0.001 versus the Ctrl group; ##*P* < 0.01 versus TGF‐β1‐stimulated cells without Spm treatment (*n* = 3). E) Representative micrographs with immunofluorescence staining of vimentin, α‐SMA, and E‐cadherin are shown in different groups. Scale bar, 10 µm. F) Kyoto Encyclopedia of Genes and Genomes (KEGG) enrichment analysis showed that differential signaling pathways were identified by the downregulated differential genes between the TGF‐β1+vehicle and TGF‐β1+Spm groups. G) Heatmap of RNA‐seq data showing the expression of fibrosis‐related genes in different groups (*n* = 3). H) qRT‐PCR showing the relative mRNA levels of fibrosis‐related genes in different groups. *P < 0.05, **P < 0.01, ***P < 0.001 versus control cells treated without Spm; #*P* < 0.05, ##*P* < 0.01, ### *P* < 0.001 versus cells‐treated with TGF‐β1 (*n* = 3). I) Cells were transfected with either of control siRNA (siNC) or siRNA specific for SMOX (siSMOX) followed by treatment with 10 ng mL^−1^ TGF‐β1 for 24 h. Western blotting and quantitative data of FN expression are presented. ***P < 0.001 versus TGF‐β1‐untreated cells; #*P* < 0.05 versus TGF‐β1‐treated cells without siSMOX (*n* = 3). J) Cells were transfected with either control vector or SMOX‐overexpression plasmid followed by treatment with 10 ng mL^−1^ TGF‐β1 for 24 h. Western blotting and quantitative data of FN expression are presented. **P < 0.01 versus TGF‐β1‐untreated cells; #*P* < 0.05 versus TGF‐β1‐treated cells without SMOX overexpression (*n* = 3).

TGF‐β1, as a key factor driving fibrosis, actively alters metabolism in diverse cell types.^[^ ^22^, ^23^ ^]^ To ascertain the direct role of spermine in ECM production in kidney cells, we applied TGF‐β1 stimulation of the mouse tubular epithelial cells (mTECs) line as an in vitro model. The results showed that the steady‐state mRNA and protein levels of SMOX were increased at both 24 and 48 hours after TGF‐β1 stimulation (**Figure** **5**A,B), accompanied with decreased cellular spermine content (Figure 5C). Furthermore, pretreatment with a relatively high dose (10 × 10^−6^ m) of spermine caused a reduction in TGF‐β1‐induced FN expression (Figure 5D). Concordantly, immunofluorescence staining showed that 10  × 10^−6^ m spermine incubation significantly inhibited vimentin and α‐SMA expression under TGF‐β1 in mTECs and restored loss of E‐cadherin at cell junctions (Figure 5E and Figure S4B, Supporting Information). To identify perturbed biologic networks and understand the biological processes and signaling pathways involved in spermine‐mediated response, mTECs were treated with TGF‐β1 in the absence or presence of spermine (10  × 10^−6^ m) for 24 h, followed by transcriptome sequencing analyses. Kyoto Encyclopedia of Genes and Genomes (KEGG) pathway analysis showed that the downregulated differential genes were mainly enriched in ECM receptor and focal adhesion signaling pathways (Figure 5F). Notably, the heat map display of differentially expressed genes also demonstrated that spermine significantly reversed the expression of fibrosis‐related genes induced by TGF‐β1 (Figure 5G). To verify these changes indeed existed, we performed qRT‐PCR on specific genes in the pathways identified by sequencing data analyses and found that pretreatment with spermine led to reduced mRNA levels of ECM‐associated factors such as *FN*, *Col I*, *α‐SMA*, connective tissue growth factor (*CTGF*), and periostin (*POSTN*) induced by TGF‐β1 (Figure 5H).

Since dysregulation of SMOX directly leads to changes in cellular spermine levels and our in vivo study showed that *Smox^+/−^* mice had less renal fibrosis compared with wild‐type controls (Figure 4), we therefore sought to determine the effect of SMOX on TGF‐β1‐induced fibrogenesis. The results showed that depletion of SMOX by siRNA significantly reduced TGF‐β1‐induced FN expression compared to scramble siRNA (siNC) (Figure 5I). Conversely, overexpression of SMOX markedly promoted FN upon TGF‐β1 stimulation (Figure 5J). These in vitro data suggest that spermine metabolism may play an antagonistic role in ECM production.

### 2.4 Exogenous Spermine or Knockdown of SMOX Improves Autophagy and Inhibits Cellular Senescence in Renal Fibrosis

**Figure 6:**
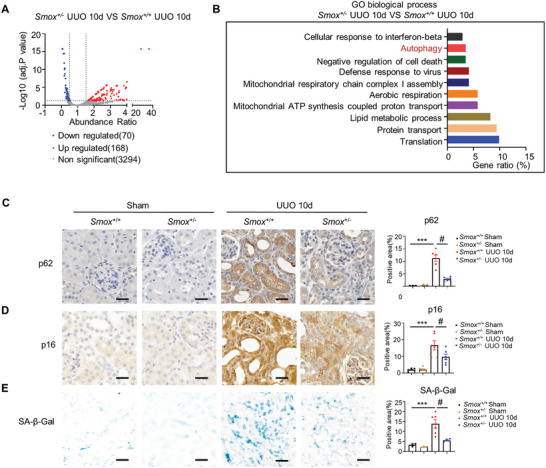
Downregulation of SMOX represses autophagy impairment and cellular senescence in renal fibrosis. A) Volcanic map on proteomic analysis shows significantly differential proteins in the kidneys of *Smox^+/+^* and *Smox^+/−^* mice with UUO (*n* = 3). B) GO enrichment analysis shows several important pathways. Data are expressed as means ± SEM. C,D) Representative micrographs and quantitative data with immunohistochemistry staining of p62 and p16 in the kidneys of *Smox^+/+^* and *Smox^+/−^* mice. Scale bar, 25 µm. ***P < 0.001 versus the *Smox^+/+^* sham; #*P* < 0.05 versus *Smox^+/+^* UUO 10d (*n* = 3–6). E) Representative micrographs and quantitative data with SA‐β‐gal activity staining in the kidneys of *Smox^+/+^* and *Smox^+/−^* mice. Scale bar, 50 µm. ***P < 0.001 versus the *Smox^+/+^* sham; #*P* < 0.05 versus *Smox^+/+^* UUO 10d (*n* = 6).

To clarify the mechanisms by which spermine metabolism regulates renal fibrosis, a proteomic study was performed on the kidney cortices from the UUO mouse model. As shown in **Figure** **6**A, a total of 70 proteins were markedly down‐regulated and 168 proteins were up‐regulated in *Smox^+/−^* mice compared to *Smox^+/+^* mice. Strikingly, gene ontology analysis confirmed that the differential proteins were significantly enriched in autophagy‐related signaling pathway (Figure 6B), demonstrating that the SMOX/spermine metabolism is closely associated with autophagy in renal fibrosis. Furthermore, *Smox* ^+/‐^ markedly abrogated increased autophagy receptor and selective substrate p62 in UUO mice compared to *Smox* ^+/+^, suggesting that SMOX restrained autophagy (Figure 6C). As both spermine and autophagy play a role in aging,^[^ ^24^, ^25^ ^]^ we examined whether the SMOX/spermine axis affects cellular senescence in the injured kidney. Significant reductions in p16‐positive senescent tubules (Figure 6D) and blue granule‐positive areas stained with Senescence‐associated β‐gal (SA‐β‐gal) (Figure 6E) were found in *Smox* ^+/‐^ UUO kidneys compared to controls. These alterations were validated in UIRI mice, where SMOX knockdown significantly improved autophagy and delayed cellular senescence in fibrotic kidneys (Figure S5A–C, Supporting Information).

**Figure 7:**
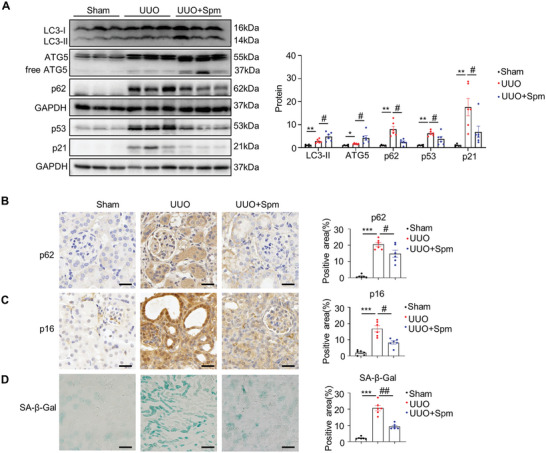
Spermine regulates the activation of autophagy and inhibits cellular senescence in renal fibrosis. A) Representative western blotting and quantitative data for indicated proteins in kidneys in different groups as indicated are shown. **P < 0.01, *P < 0.05 versus sham; #*P* < 0.05 versus UUO mice treated with vehicle (*n* = 6). B,C) Representative micrographs and quantitative data with immunohistochemistry staining of p62 and p16 in different groups. Scale bar, 25 µm. ***P < 0.001 versus the sham; #*P* < 0.05 versus UUO mice treated with vehicle (*n* = 6). D) Representative micrographs and quantitative data with SA‐β‐gal activity staining in different groups. Scale bar, 50 µm. ***P < 0.001 versus the sham; ##*P* < 0.01 versus UUO mice treated with vehicle (*n* = 6).

We next examined renal autophagy and cellular senescence in UUO and UIRI mice supplemented with spermine. As shown in **Figure** **7**A, compared with vehicle‐treated UUO mice, spermine administration significantly increased LC3‐II levels, ATG5, and concomitant degradation of p62 in obstructed kidneys, indicating the activation of autophagy. Meanwhile, spermine reduced UUO‐induced expression of senescence markers p53 and p21 in the kidneys. The p62 and p16 staining, as well as the SA‐β‐gal positive area, which were markedly induced in the UUO kidney, were also abrased by spermine (Figure 7B–D). Spermine supplementation in UIRI mice resulted in similar outcomes (Figure S5D–G, Supporting Information). Collectively, these data demonstrate that impaired autophagy and accelerated cellular senescence occur in fibrotic kidneys, which is alleviated by SMOX knockdown or spermine supplementation.

### 2.5 The Interaction between ATG5 and SMOX Affects the Spermine Levels in Kidney

**Figure 8:**
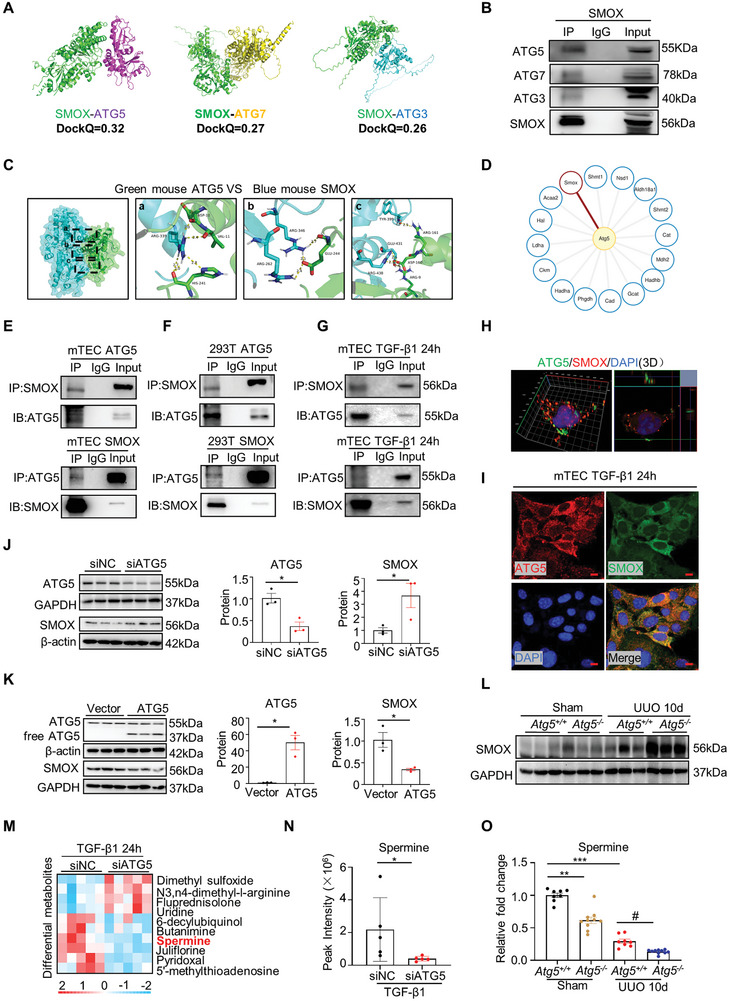
ATG5 interacts with and suppresses the expression of SMOX. A) Images represent the 3D structure and the potential binding of SMOX (green) proteins and ATG5 (purple), ATG7 (yellow), and ATG3 (blue) respectively. DockQ represents the confidence of the potential binding. B) Co‐immunoprecipitation of SMOX and autophagy‐related protein ATG5, ATG7, ATG3. C) Images represent the 3D structure of ATG5 (green) and SMOX (blue) proteins, and the dotted box indicates the binding site of ATG5 and SMOX protein. D) mTECs were transfected with ATG5 overexpression plasmid, and cell lysate was immunoprecipitated (IP) with anti‐ATG5 antibody followed by examination the affected proteins by mass spectrometry. E–G) mTECs or 293T cells were treated with or without TGF‐β1 as indicated. Coimmunoprecipitation of ATG5 and SMOX. H) Double immunofluorescence staining of ATG5 and SMOX in 293T cells. Scale bar, 5 µm. I) Double immunofluorescence staining of ATG5 and SMOX in TGF‐β1‐treated mTECs. Scale bar, 10 µm. J) Representative western blotting and quantitative data for protein levels of SMOX and ATG5 in mTECs transfected with either control siRNA (siNC) or siRNA specific for ATG5 (siATG5). *P < 0.05 versus siNC (*n* = 3). K) Representative western blotting and quantitative data for SMOX and ATG5 protein levels in mTECs transfected with ATG5 empty vector or overexpression plasmid. *P < 0.05 versus empty vector (*n* = 3). L) Western blotting showing SMOX protein expression in the kidneys of *Atg5^+/+^* and *Atg5^−/−^* mice with or without UUO. M,N) mTECs were transfected with either negative control siRNA (siNC) or siRNA specific for ATG*5* (siATG5) followed by treatment with 10 ng mL^−1^ of TGF‐β1 for 24 h. M) The heatmap shows the different metabolites in cells by non‐target metabolomics. N) The graphical representation shows the relative level of intracellular spermine. *P < 0.05 versus siNC (*n* = 5). O) Graphical representation showing the relative spermine levels by ELISA in the kidneys of *Atg5^+/+^* and *Atg5^−/−^* mice with or without UUO. **P < 0.01, ***P < 0.001 versus *Atg5^+/+^* sham controls; #*P* < 0.05 versus *Atg5^+/+^* UUO mice (*n* = 8). Data are expressed as means ± SEM.

An interaction loop has been reported between polyamine metabolism and autophagy,^[^ ^26^ ^]^ we therefore sought to unravel the importance of this crosstalk in kidney fibrogenesis. AlphaFold 2.3.0 multimers were used to predict protein–protein interactions, yielding reliable DockQ values, which showed the highest DockQ for SMOX‐ATG5 interaction (**Figure** **8**A). Coimmunoprecipitation in mTECs confirmed that SMOX did have a strong interaction with ATG5 but a weak interaction with ATG3 and ATG7 (Figure 8B). Meanwhile, the crystal structure of the protein was analyzed by molecular docking, and it was found that ATG5 and SMOX proteins derived from human and mouse had multiple potential binding sites (Figure 8C and Figure S7A, Supporting Information). Further, we utilized coimmunoprecipitation in combination with mass spectrometry to detect what ATG5‐binding partners are in mTECs. Consistently, we were able to find that these ATG5‐interacting proteins included 16 amino acid metabolism‐related proteins, in which SMOX was present (Figure 8D). The ATG5‐SMOX interaction was further verified by co‐immunoprecipitation experiments in mTECs and HEK‐293T cells following overexpression of both proteins under basal conditions (Figure 8E,F) or exposure to TGF‐β1 (Figure 8G). Since the most important non‐covalent interaction in biomolecules is hydrogen bonding,^[^ ^27^ ^]^ an ATG5 multisite‐mutated (MMut) plasmid based on the potential binding sites of hydrogen bonds was constructed and transfected into mTECs, with wild‐type ATG5 as a control. Unlike wild‐type ATG5, the ATG5 MMut protein failed to interact with SMOX, indicating that ATG5 and SMOX interact through these predicted binding sites (Figure S7B, Supporting Information). Additionally, based on the prediction that ATG5 GLU244 binds to 3 loci of SMOX via hydrogen bonds, we examined the possibility of GLU244 as a potential key binding site. However, the results showed that the E244A single‐site mutation (SMut) of ATG5 had no significant effect on the interaction between ATG5 and SMOX (Figure S7C, Supporting Information), suggesting that the interaction between the two does not mainly depend on this site, but on multiple sites. Regardless, colocalization of ATG5 and SMOX was observed by immunofluorescent staining in 293T cells (Figure 8H) and TGF‐β1‐stimulated mTECs (Figure 8I), as well as in the kidneys of CKD patients (Figure S7D, Supporting Information).

The clear evidence of the combination of the ATG5 and SMOX drove us to further observe the cross‐talk between their expression and function. Strikingly, downregulating ATG5 increased the protein expression of SMOX in mTECs (Figure 8J), while, overexpressed ATG5 decreased SMOX expression (Figure 8K). Further, in UUO model, it was also found that SMOX expression in the kidneys of *Atg5^−/−^* mice was significantly increased compared with *Atg5^+/+^* mice (Figure 8L). Collectively, both in vitro and in vivo results indicate that ATG5 negatively regulates SMOX protein expression. To this end, mTECs were transfected with siRNA against ATG5 or control siRNA, followed by metabolomic analysis to measure intracellular spermine levels in response to TGF‐β1 stimulation. Among differential metabolites, downregulation of ATG5 significantly decreased TGF‐β1‐induced spermine content compared to control siRNA cells (Figure 8M,N). In vivo, we generated tubule‐specific *Atg5*‐knockout mice using a Cre‐LoxP recombination system to better elucidate the effect of ATG5 expression on spermine metabolism in renal TECs. The spermine contents in the kidneys of *Atg5^+/+^* and *Atg5^−/−^* mice, with or without UUO, were detected by ELISA. As shown in Figure 8O, in the sham‐operated group, *Atg5^−/−^* mice exhibited lower spermine contents than their wild‐type littermates, denoting that impaired autophagy may lead to rapid spermine consumption under physiological conditions. Consistent with our observation above (Figure 1I,J), spermine levels were reduced in *Atg5^+/+^* UUO mice relative to sham‐operated *Atg5^+/+^* or *Atg5^−/−^* mice, and this response was significantly aggravated in *Atg5^−/−^* mice after UUO, suggesting that inhibition of autophagy can further exacerbate spermine decline under diseased states. On the other side, the effect of spermine was achieved by enhancing autophagy. Spermine supplementation in *Atg5^+/+^* UUO or UIRI mice could reduce the accumulation of collagen fibers in the kidneys by half, while this effect disappeared in *Atg5^−/−^* mice receiving the same treatment (Figure S6, Supporting Information). And the ATG5/SMOX interaction may be involved in the occurrence and development of renal fibrosis through regulating spermine content.

### 2.6 Spermine Inhibits TGF‐β1‐Induced Fibroblast Activation and Proliferation

**Figure 9:**
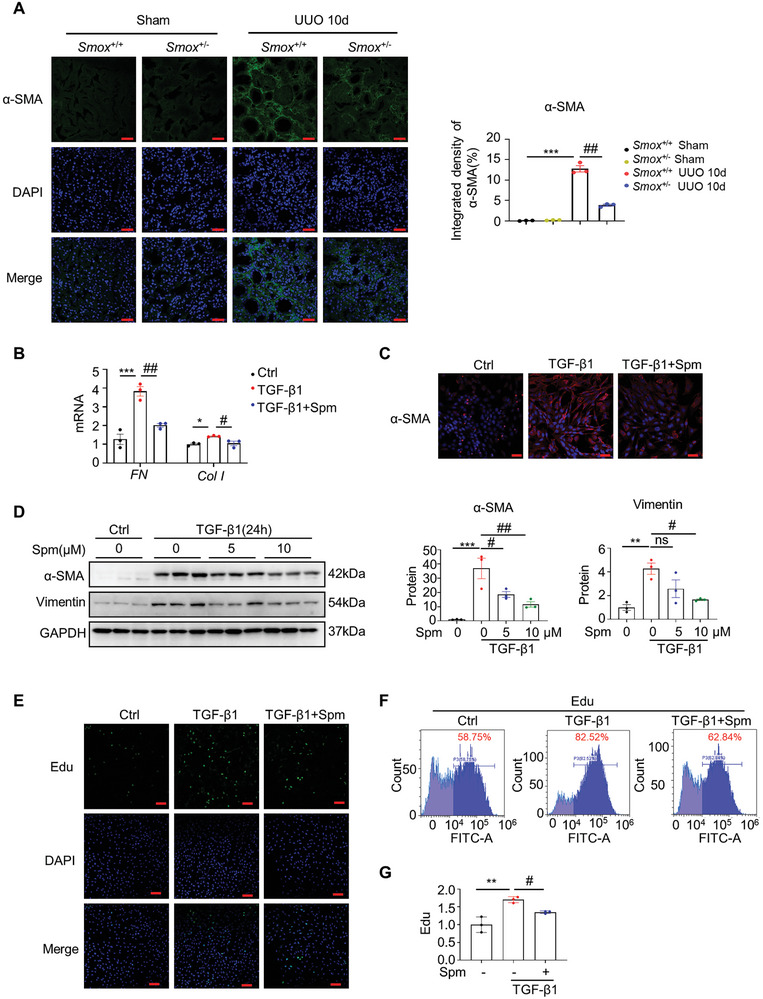
Spermine inhibits TGF‐β1‐induced fibroblast proliferation and activation. A) Representative micrographs and quantitative data with immunofluorescence staining of α‐SMA in the kidneys of *Smox^+/+^* and *Smox^+/−^* mice. Scale bar, 50 µm. ***P < 0.001 versus the *Smox^+/+^* sham; ##*P* < 0.01 versus *Smox^+/+^* UUO 10d (*n* = 3). B) NRK‐49F cells were preincubated with 10 × 10^−6^ m spermine (Spm) for 12 h and then treated with TGF‐β1 (5 ng mL^−1^) for 24 h and subjected to qRT‐PCR detection. C) Representative micrographs with immunofluorescence staining of α‐SMA are shown. Scale bar, 10 µm. DAPI denotes the nuclei. D) Western blotting and quantitative data of α‐SMA and vimentin in different groups as indicated are shown. E–G) Representative micrographs with immunofluorescence staining and flow cytometry plots showing the mean fluorescence intensity of EdU in different groups. Scale bar, 10 µm. Data are expressed as means ± SEM, *P < 0.05, **P < 0.01, ***P < 0.001 versus Ctrl; #*P* < 0.05, ##*P* < 0.01 versus TGF‐β1; ns, means no significant (*n* = 3).

Fibroblasts are important effector cells in the process of renal fibrosis. We noted that SMOX knockdown markedly attenuated UUO‐induced expression of the marker α‐SMA in activated fibroblasts (**Figure** **9**A). We thus evaluated whether exogenous spermine could suppress fibroblast activation. To this end, cultured fibroblasts (NRK‐49F) were incubated with TGF‐β1 in the absence or presence of spermine. As shown in Figure 9B, spermine (10 × 10^−6^ m) repressed TGF‐β1‐induced mRNA expression of *α‐SMA* and *Col I*. Immunofluorescent staining also showed that TGF‐β1‐triggered α‐SMA expression was reduced by treatment with spermine (Figure 9C). Further, spermine supplementation inhibited TGF‐β1‐induced expression of α‐SMA and vimentin (Figure 9D). Collectively, these data suggest the ability of spermine to abolish the myofibroblastic activation of renal fibroblasts.

To investigate the effect of spermine on TGF‐β1‐induced fibroblast proliferation, we used an EdU kit to measure the percentage of EdU‐positive NRK‐49F cells. As shown in Figure 9E, the number of EdU‐positive cells was dramatically increased upon TGF‐β1 stimulation, however, spermine obviously attenuated this response. Flow cytometry analysis revealed that under TGF‐β1 stimulation, the percentage of proliferating NRK‐49F cells increased from 58.75% in the ground state to 82.52%, while in spermine‐treated cells, this stimulating effect was diminished to 62.84% (Figure 9F,G), suggesting that spermine also alleviated renal fibroblast proliferation.

## 3 Discussion

**Figure 10:**
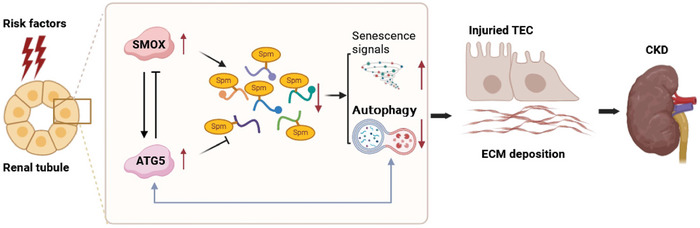
Schematic illustration of the potential mechanism of SMOX/spermine axis against renal fibrosis. Created with BioRender.com.

In this study, we demonstrate that SMOX protein expression was increased predominantly in tubular epithelium in fibrotic kidneys of patients and mice. Upregulated renal SMOX was positively correlated with kidney fibrosis and function decline in CKD patients. Notably, supplementation with spermine or genetically deficient *SMOX* improved autophagy, repressed cell senescence, and alleviated renal fibrosis. Further, ATG5 physically interacted with SMOX that affected cellular spermine content. Our findings demonstrate a spermine oxidase/spermine axis that links polyamine metabolism to kidney fibrogenesis and suggest that targeting spermine pathway is a novel potential therapeutic avenue for CKD (**Figure** **10**).

SMOX, a key catabolic enzyme in polyamine metabolism, was easily induced by various stimuli, and upregulation of SMOX reduced intracellular spermine pools.^[^ ^12^, ^28^ ^]^ However, the changes in SMOX expression and spermine content in experimental kidney disease remain controversial. It has been suggested that *Smox* mRNA level and spermine content were significantly decreased in fibrotic kidneys at day 21 after IRI,^[^ ^29^ ^]^ while in another study, *Smox* mRNA level was markedly increased on day 14 after UUO without affecting spermine levels in the kidneys.^[^ ^30^ ^]^ Interrogation of available single‐cell RNA‐seq datasets (http://humphreyslab.com), on the other hand, a recent study demonstrated a distinct proportion of tubular epithelial cells with highly and broadly upregulated transcription level of *Smox* in UUO and IRI mouse kidneys.^[^ ^31^ ^]^ Our data were consistent with these findings and showed that SMOX was noticeably increased at both mRNA and protein levels in mouse fibrotic kidney tissues and that SMOX was predominantly expressed in tubular epithelium in fibrotic kidneys of patients and mice. Further, our results extend previous studies showing a reduction of spermine content in the kidneys of UUO and IRI mice,^[^ ^29^, ^32^ ^]^ as well as decreased renal tubular epithelial cellular levels of spermine upon TGF‐β1 exposure.^[^ ^32^ ^]^ In vivo, we also found that the levels of spermidine were significantly increased in the kidneys of both fibrosis models, while putrescine was elevated only in UUO kidneys. The dynamic changes in SMOX and polyamine levels during renal fibrosis may differ depending on the model and its observation time points. Since spermine is the end‐product of the polyamine metabolic pathway, reduced spermine content in fibrotic kidneys may be caused by multiple processes, including enzymes that related to spermine metabolism. As such, our study focused on increased SMOX promoting spermine catabolism in fibrotic kidneys. Apart from endogenous metabolism, food consumption, and intestinal microbiota are the other two sources of spermine.^[^ ^10^ ^]^ However, CKD patients often exhibit intestinal flora disorders and impaired intestinal barrier,^[^ ^33^ ^]^ which may also lead to reduced intake of spermine. So far, it appears uncertain fibrosis‐associated changes in SMOX expression and spermine content, more studies are needed to confirm these findings.

Using proteomics, here we found that the biological process of autophagy was affected in fibrotic kidneys of *Smox* knockdown mice, prompting us to further focus on the autophagy signaling pathway in the mechanistic exploration of SMOX. Autophagy, as an important regulator of cellular metabolism, is essential for maintaining kidney homeostasis, structure, and function.^[^ ^34^ ^]^ Furthermore, the reaction products spermine and spermidine of SMOX are potent autophagy agonists.^[^ ^25^, ^35^, ^36^, ^37^ ^]^ Yet, a direct relationship between SMOX and autophagy has not been reported. Our results demonstrated that either spermine administration or genetically deficient SMOX enhanced UUO‐induced autophagy and attenuated renal fibrosis, reinforcing the SMOX/Spermine axis and autophagy interaction as the core mechanism of CKD. Cellular senescence is stress response essential for homeostasis.^[^ ^38^ ^]^ Tubular epithelial senescent cells exhibit a secretory phenotype, which can drive chronic inflammation and accelerate the progression of renal fibrosis.^[^ ^39^ ^]^ Studies have revealed that spermine is significantly decreased in kidneys of aged rats,^[^ ^40^ ^]^ and exogenous polyamine supplementation delays age‐related diseases.^[^ ^41^, ^42^, ^43^ ^]^ Moreover, diets rich in polyamines reduce age‐related glomerulosclerosis and mortality in elderly mice.^[^ ^44^ ^]^ Thus, it is conceivable that action of spermine in vivo may go beyond the simple activation of autophagy. Consistent with previous studies showing that spermine administration reduced renal p53 and p21 expression, as well as the activity of SA‐β‐gal in UUO mice. Collectively, our findings indicated that spermine protected against renal fibrosis through enhancing autophagy activity and inhibiting cell senescence.

It is worth noting that autophagy has an essential role in maintaining cellular polyamine metabolic homeostasis.^[^ ^26^ ^]^ The readily interaction between SMOX and ATG5 was identified by co‐immunoprecipitation and mass spectrometry. Furthermore, the structure of the mouse SMOX sequence with 34‐268 and 309‐544 site features for the oxidase site, which overlaps with the ATG5 and SMOX binding sites analyzed with the interPro protein structure domain database (https://www.ebi.ac.uk/interpro/search/sequence/). Notably, downregulation of ATG5 increased SMOX protein expression, whereas overexpression of ATG5 reduced SMOX expression. Thus, we speculate that the interaction between ATG5 and SMOX may positively affect SMOX function, thereby resulting in altered spermine levels. To explore whether autophagy is involved in spermine regulation, we suppressed autophagy by depleting ATG5, a key regulation of autophagy, in renal TECs. Our in vivo and in vitro results revealed that ATG5 knockdown aggravated a decrease in spermine during renal fibrosis. Subsequently, the protective role of spermine supplementation was disrupted in ATG5‐deficient mice, which in part indicates that ATG5‐mediated autophagy is required for the protective effects of spermine against renal fibrosis. Collectively, these results indicate that the SMOX/spermine axis coordinates ATG5‐mediated autophagy to orchestrate renal fibrosis.

Injured tubular cells produce profibrotic factors, such as TGF‐β1, leading to fibroblast activation and renal fibrosis.^[^ ^45^ ^]^ Our results demonstrated that spermine supplementation effectively inhibited extracellular matrix production by mTECs and fibroblasts. Additionally, SMOX knockdown in mice markedly attenuated the UUO‐induced fibroblast accumulation and depletion of SMOX in mTECs significantly reduced TGF‐β1‐induced FN expression. Notably, increased expression of SMOX was only observed in renal TECs but not in fibroblasts (data not shown). Therefore, SMOX‐mediated spermine reduction is most likely to occur primarily in TECs, thereby promoting epithelial phenotype loss and in vivo fibroblast activation, ultimately leading to ECM accumulation and renal fibrosis.

The present study has several limitations and leaves questions unanswered. First, our SMOX global knockdown murine model is not restricted to renal tubular epithelial cells, further study is needed to elucidate the context and cell‐type‐dependent role of SMOX by using a cell‐specific inducible model. Second, although we confirmed the interaction of SMOX with the potential binding sites of ATG5, additional exploration is warranted to ascertain the exact binding sites within ATG5. Thirdly, beyond autophagy and cellular senescence, whether other signaling pathways are involved in the SMOX/Spermine axis’ effects in renal fibrosis as well as their complex interrelated mechanisms remain to be explored.

In conclusion, we have identified the protective roles of downregulated SMOX expression and increased spermine content in TGF‐β1‐induced fibrogenesis in vitro and kidney fibrosis in vivo, using two independent models of kidney fibrosis. Furthermore, we demonstrated that ATG5 physically interacted with SMOX to modulate the occurrence and development of renal fibrosis through maintaining intracellular spermine levels. Our study suggests that targeting spermine metabolism may have therapeutic benefits for patients with renal fibrosis.

## 4 Experimental Section

### Human Kidney Samples

Human kidney specimens collected from patients with CKD in the First Affiliated Hospital of Sun Yat‐Sen University, with written informed consent were analyzed. The clinical characteristics of those patients at the initial biopsy were described in Table S1 (Supporting Information). The severity of renal fibrosis was determined by the Masson trichrome staining according to the previous report.^[^ ^46^ ^]^ The renal fibrosis degree was classified according to Banff classification: grade 0 was no fibrosis, grade 1 was mild fibrosis (collagen fibers accounted for less than 25% of the kidney tissue), grade 2 was moderate fibrosis (accounted for 25%−50%), and grade 3 was severe fibrosis (accounted for more than 50%).^[^ ^47^ ^]^ The linear regression between SMOX expression and renal fibrosis was evaluated according to previous reports.^[^ ^48^ ^]^ Non‐tumor renal tissues from eight patients with renal cell carcinoma were used as control. This study was approved by the First Affiliated Hospital of Sun Yat‐Sen University Institutional Review Board (project number: (2023)050).

### Mice and Animal Models

Male wild‐type C57BL/6 mice and Smox‐KO mice(Strain NO.T027342)were purchased from GemPharmatech (Nanjing, Jiangsu, China). *Atg5* ^flox/flox^ mice were purchased from the RIKEN BioResource Center (Tsukuba, Ibaraki, Japan). Proximal tubule‐specific *Atg5^−/−^* mice were generated by crossing an *Atg5* ^flox/flox^ mouse with a Kap‐cre mouse, as described previously.^[^ ^49^ ^]^ The Kap‐cre mice expressing Cre recombinase under the control of the Kap promoter were from the Jackson Laboratory (Bar Harbor, Maine, USA). Genotyping was identified by the polymerase chain reaction (PCR) from genomic DNA obtained from tail biopsies, the primer sequences were shown in Table S2 (Supporting Information). Mice were maintained under pathogen‐free facility of the Laboratory Animal Center, Sun Yat‐Sen University. Animal experiments were approved by the Ethics Committee for the Use of Experimental Animals of Yat‐Sen University (project number: 2022001708).

Mice aged 8–12 weeks were used for experiments. UUO and UIRI surgery were performed as described previously.^[^ ^46^, ^49^ ^]^ Briefly, for the UUO injury model, mice were anesthetized, and the abdomen was opened with a midline incision, the left ureter was exposed and ligated twice with 5‐0 silk sutures. Sham‐operated mice had their left ureters exposed and manipulated but ligated. Mice were sacrificed 7 or 10 d after operation. For the UIRI model, mice were anesthetized and the left renal pedicle was clamped for 45 min at 37 °C through a left abdominal incision. Mice were sacrificed 7 or 14 d after reperfusion. For some experiments, 1 mg kg^−1^ of spermine (Cat#S4264, Sigma‐Aldrich, St Louis, MO, USA) was injected intraperitoneally as indicated in the results section. Blood and kidneys were collected for analysis.

### Cell Culture and Treatment

Mouse kidney proximal tubular epithelial cells (mTEC, CRL‐3361), normal rat kidney fibroblasts (NRK‐49F, CRL‐1570), and human 293T (293T, CRL‐11268) were purchased from American Type Culture Collection. mTEC cells and 293T cells were cultured in Dulbecco's modified Eagle medium/Nutrient Mixture F‐12 (Cat #C11330500BT, Gibco, Thermo Fisher Scientific, Halethorpe, MD), supplemented with 10% fetal bovine serum (Cat#10099‐141C, Gibco). NRK‐49F were maintained in Dulbecco's Modified Eagle Medium (Cat#C11995500BT, Gibco) with 10% fetal bovine serum. Cells were pretreated with or without spermine (5 × 10^−6^ or 10 × 10^−6^ m) for 12 h, followed by treatment with 10 ng mL^−1^ TGF‐β1 (Cat#240‐B, R&D systems, Minneapolis, MN, USA) for an additional 24 h. The siRNA transfection was performed using Lipofectamine 2000 (Cat#11668019, Invitrogen, Thermo Fisher Scientific, Halethorpe, MD) or plasmids were introduced into cells using PolyJet regent (Cat#SL100688, SignaGen Laboratories, USA), according to the manufacturer's instructions. The MMut plasmid has mutated a total of 10 amino acid binding sites of ATG5 that potentially bind to SMOX, including T2A, D3A, R9A, D10A, V11A, D160A, R161A, H241A, E244A, and M246A, and The SMut plasmid (mutated with E244A) were used for transfection and coimmunoprecipitation of mTECs. The information of targeted siRNA and plasmids are shown in Table S3 (Supporting Information).

### Measurement of Spermine Concentration

ELISA assay was performed to determine the amount of spermine in the kidneys or cells by using Mouse spermine ELISA kit (Cat# LV30695, Animalunion, Shanghai, China) according to the manufacturer's instructions. Briefly, the kidneys or cells were homogenized with PBS and centrifuged at 12 000 rpm for 20 min at 4 °C. The supernatant was collected to examine the production of spermine, and protein content of the supernatant quantified by BCA assay was used to correct spermine concentration.^[^ ^50^ ^]^ Detection of renal tissue spermine by mass spectrometry is commissioned by a platform according to standard procedures (LipidALL Technologies Co., China). The content of spermine was analyzed by Jasper HPLC‐SCIEX 4500 MD. The chromatography conditions were as follows: Waters Acquity UPLC SS‐T3 (3×100 mm, 1.8 µm), A: 0.1% formic acid, mobile phase B: Acetonitrile.

### Untargeted Metabolomics Analysis

mTECs were transfected with either negative control or ATG5 siRNA as described above. After exposure to TGF‐β1 (10 ng mL^−1^) for 24 h, cells were collected for untargeted metabolomics analysis with liquid chromatography/mass spectrometer (Q Exactive HF) (Thermo Fisher Scientific, USA) via BGI Genomics platform following a standard method (BGI, Shenzhen, China). In order to increase the metabolite coverage, samples were analyzed under both positive and negative ionization modes. The raw metabolome data pretreatment, including peak alignment, peak extraction, and compound identification were processed using Compound Discoverer 3.1 (Thermo Fisher Scientific, USA), followed by metabolite annotation and statistical analysis. Metabolomics data have been submitted to MetaboLights (www.ebi.ac.uk/metabolights/MTBLS8529).

### Transcriptomic Analysis

RNA isolation, library construction, and sequencing were performed by Novogene (Beijing, China). Sequencing was performed on an Illumina NovaSeq 6000. The sequencer images were transformed into reads through base recognition analysis and clean reads were obtained after raw data filtering, error data checking, and GC‐content distribution checking. Reference genome and gene model annotation files were downloaded from genome website directly. Index of the reference genome was built using Hisat2 (v2.0.5) and paired‐end clean reads were aligned to the reference genome. Through the Novogene bioinformatics platform, differentially expressed genes, KEGG pathway enrichment analysis and heatmap analysis were analyzed. For the data generated in this study, the RNA sequencing data has been submitted to National Center for Biotechnology Information (NCBI) Sequence Read Archive (SRA) database with the identifier PRJNA 1015396.

### Mass Spectrometry‐Based Protein Identification

After co‐immunoprecipitation, proteins were separated by SDS‐PAGE, extracted from gel slices, and digested with trypsin to extract the peptide. The peptide samples were redissolved with mobile phase A (2% Acetonitrile, 0.1% formic acid), centrifuged at 20 000 *g* for 10 min, and then the supernatant was taken into the separation by Thermo UltiMate 3000 UHPLC. Spectra of peptide fragments were analyzed by Q‐Exactive HF X (Thermo Fisher Scientific, USA) and matched to the theoretical spectra. After quality control and filtering, protein identification results were obtained.

### Statistical Analysis

Statistical processing was performed using GraphPad Prism Software (version 8.0). Quantitative values of all experimental data were expressed as Mean ± standard error of the mean (SEM). Unpaired Student's t‐test (two‐tailed) was conducted for comparison between two groups, and one‐way ANOVA with Tukey's test was used for comparison between multiple groups. *P* < 0.05 indicated a statistically significant difference.

## Conflict of Interest

The authors declare no conflict of interest.

## Author Contributions

D.L., X.H.L., and H.Y.L. contributed equally to this work. D.L. and X.H.L. performed major experiments and wrote the original draft. Y.T.W. and H.Y.L. performed the experiment design and data analysis. H.Y.L., S.M.J., G.L.L., and Y.P.X. performed the animal experiments and kidney histologic examination. K.F.W., X.R.D., and Q.H.L. performed project administration and manuscript preparation. W.C., Y.Z., and H.P.M. designed the study and revised the manuscript. All authors read and approved the final manuscript.

## Supporting information

Supporting Information

Supporting Information

## Data Availability

The data that support the findings of this study are openly available in National Center for Biotechnology Information (NCBI) Sequence Read Archive (SRA) database at https://dataview.ncbi.nlm.nih.gov/object/PRJNA1015396, reference number PRJNA 1015396.
