# Supplementary material for: The Spermine Oxidase/Spermine Axis Coordinates ATG5‐Mediated Autophagy to Orchestrate Renal Senescence and Fibrosis
Source: Adv Sci (Weinh). 2024 May 22;11(29):2306912. doi: 10.1002/advs.202306912 (PMC11304251; doi:10.1002/advs.202306912)
Supplement: Supplementary file 1 — Supporting Information [file ADVS-11-2306912-s002.docx]

Supporting Information

**The Spermine Oxidase/Spermine Axis Coordinates ATG5-Mediated Autophagy to Orchestrate Renal Senescence and Fibrosis**

*Dan Luo^1,2§^, Xiaohui Lu^1§^,* *Hongyu Li^1§^, Yi Li^1^, Yating Wang^1^, Simin Jiang^1^, Guanglan Li^1^, Yiping Xu^1^, Kefei Wu^1^, Xianrui Dou^2^, Qinghua Liu^1^, Wei Chen**^1^*, Yi Zhou^1^*, Haiping Mao^1^**

^1^Department of Nephrology, The First Affiliated Hospital, Sun Yat-sen University, NHC Key Laboratory of Clinical Nephrology, Guangdong Provincial Key Laboratory of Nephrology, Guangzhou, Guangdong, 510080, China

^2^Department of Nephrology, Shunde Hospital, Southern Medical University (The First People’s Hospital of Shunde), Foshan, Guangdong, 528308, China

**
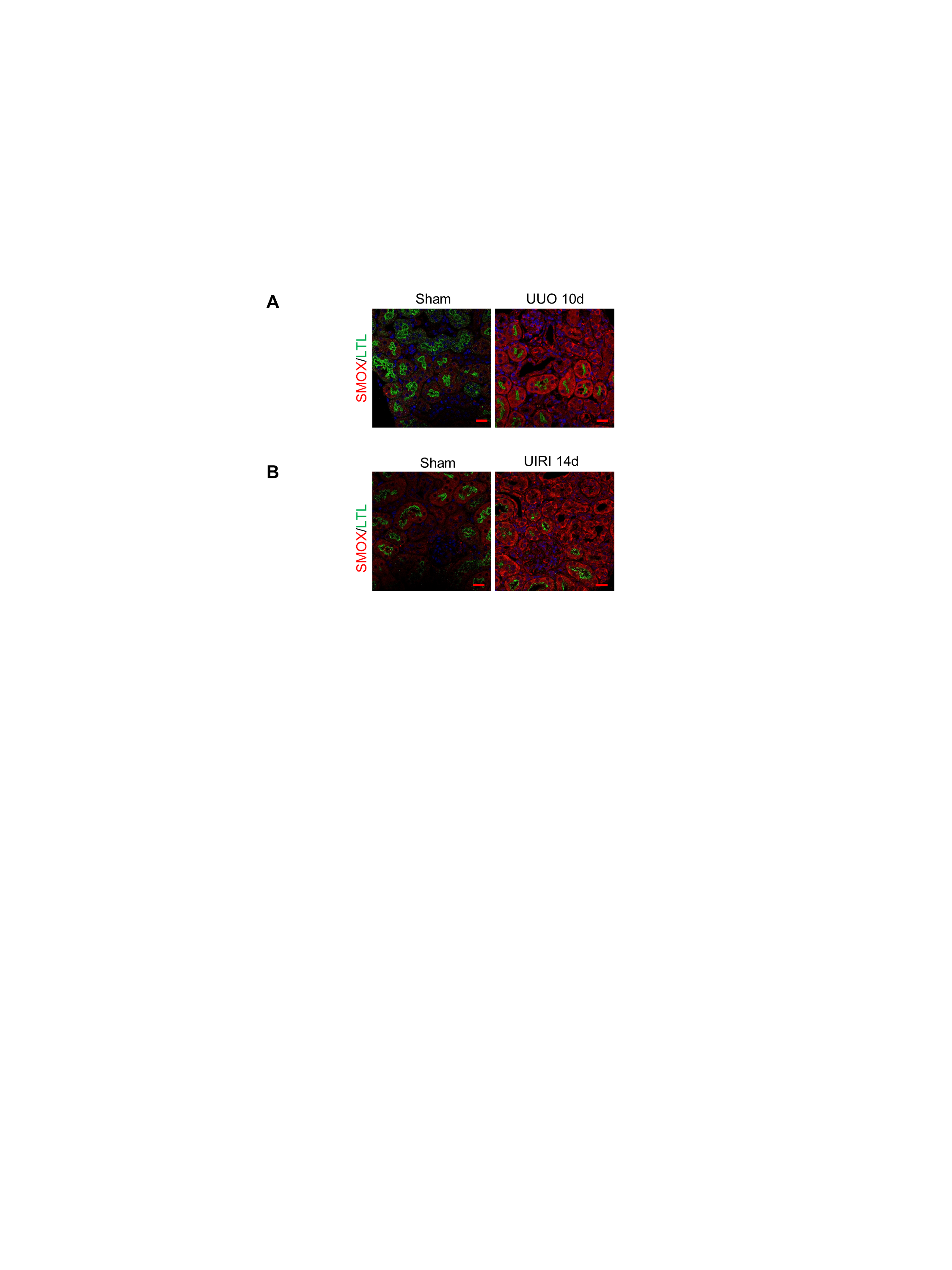
**

**Figure S1** The distribution of SMOX in fibrotic kidneys of mice. Colocalization of SMOX (red) with LTL (green), a marker of proximal tubular epithelial cells, in the kidney tissues of **A**) UUO and **B**) UIRI mice. Scale bar, 20 µm.


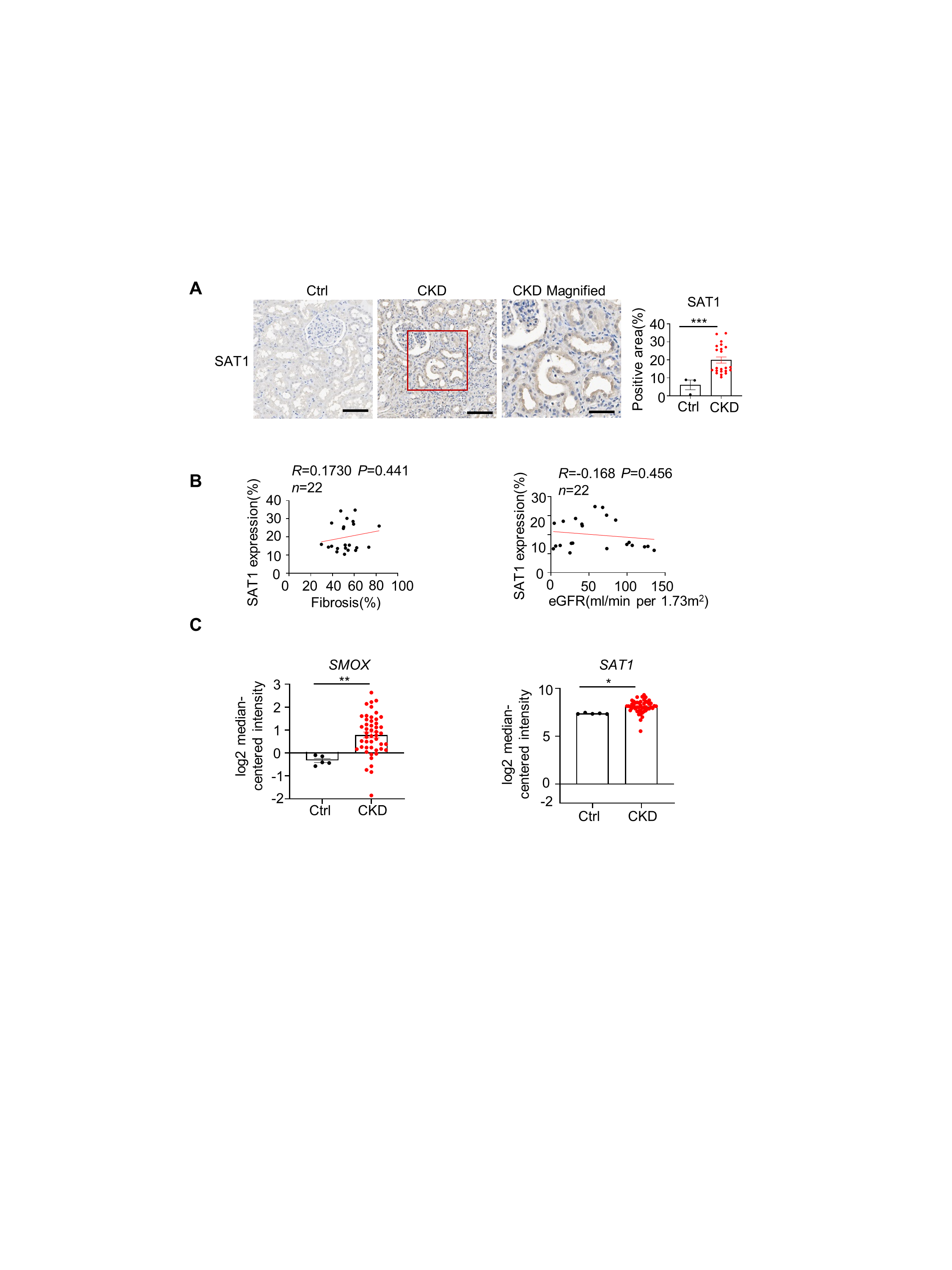


**Figure S2** Increased SAT1 expression in CKD patients. **A**) Representative images of SAT1 staining in kidney tissue CKD patients. Non-tumor kidney tissues from patients with renal carcinoma were used as controls (Ctrl). Scale bar, 100 µm. Graphical representation showing the relative abundance of SAT1 expression in the kidneys of Ctrl and CKD patients. **B**) The correlations between the positive area of SAT1 staining and renal fibrosis and eGFR in CKD patients. The *P* value, number of patients (n), and Spearman correlation coefficient (R) are indicated on the graph. **C**) The renal mRNA levels of *SMOX* and *SAT1* in healthy controls (n = 5) and CKD patients (n = 48) were analyzed from the Nephroseq database (<http://www.nephroseq.org>) (GSE66494). Data are expressed as means ± SEM, **P* < 0.05, ***P* < 0.01，****P* < 0.001 versus healthy controls.


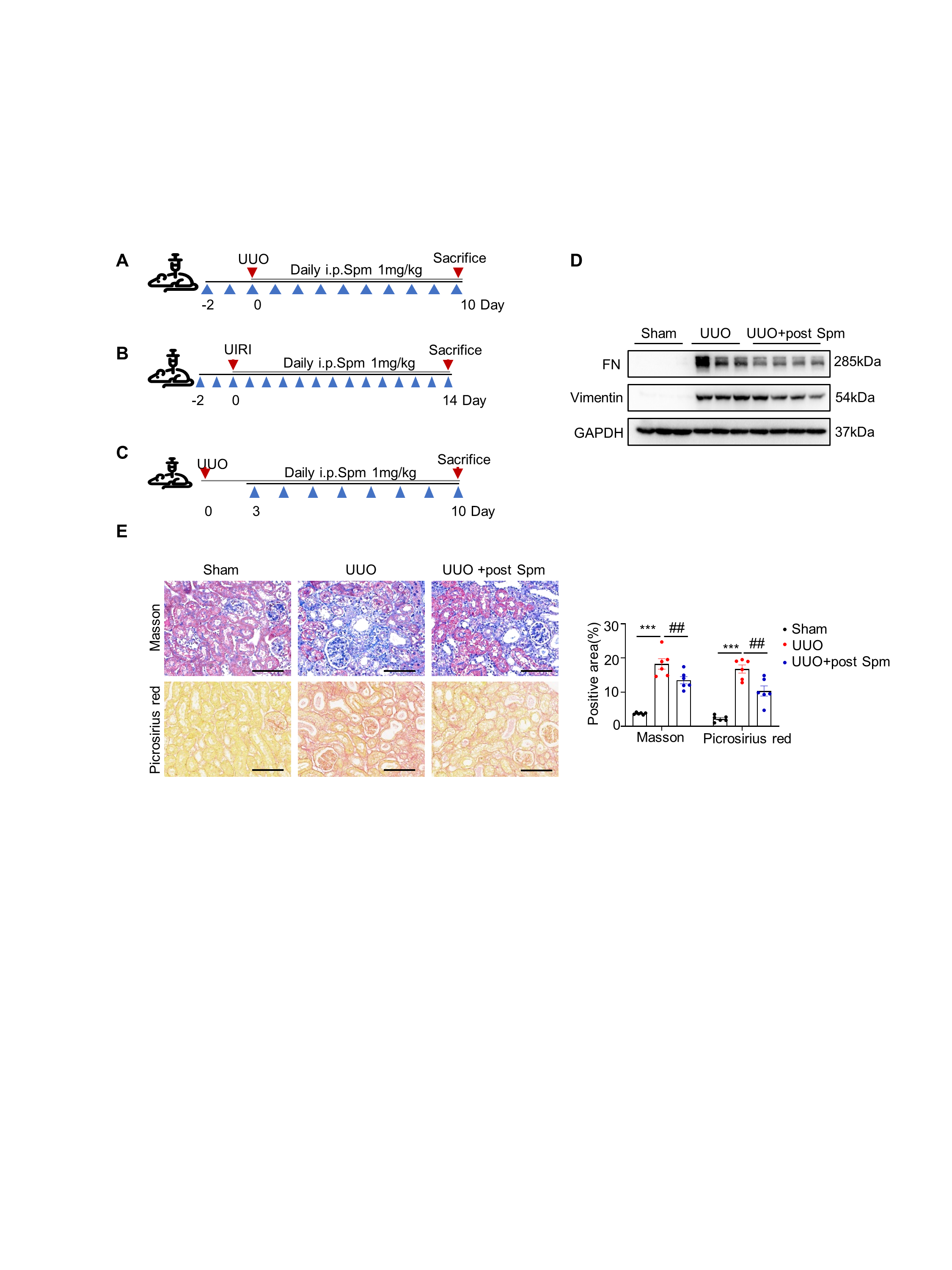


**Figure S3** Application scheme and therapeutic effect of spermine in renal fibrosis. **A, B**) The experimental design: red arrowheads indicate the timing of **A**) UUO or **B**) UIRI and sacrifice. Blue arrowheads represent the daily intraperitoneal injection of spermine (Spm). **C**) The experimental design for spermine post-treatment for renal UUO: Red arrowheads indicate the timing of UUO and sacrifice. Blue arrowheads represent the daily intraperitoneal injection of spermine (Spm). **D**) Representative western blotting showing renal expression of Fibronectin (FN) and vimentin in different groups. **E**) Masson trichrome and Sirius red staining images in various groups as indicated are shown. Scale bar, 100 µm. Quantitative determination of collagen deposition area based on Masson’s trichrome and Sirius red staining in different groups. Data are expressed as means ± SEM, ****P* < 0.001 versus sham; ##*P* < 0.01 versus UUO (n = 6).


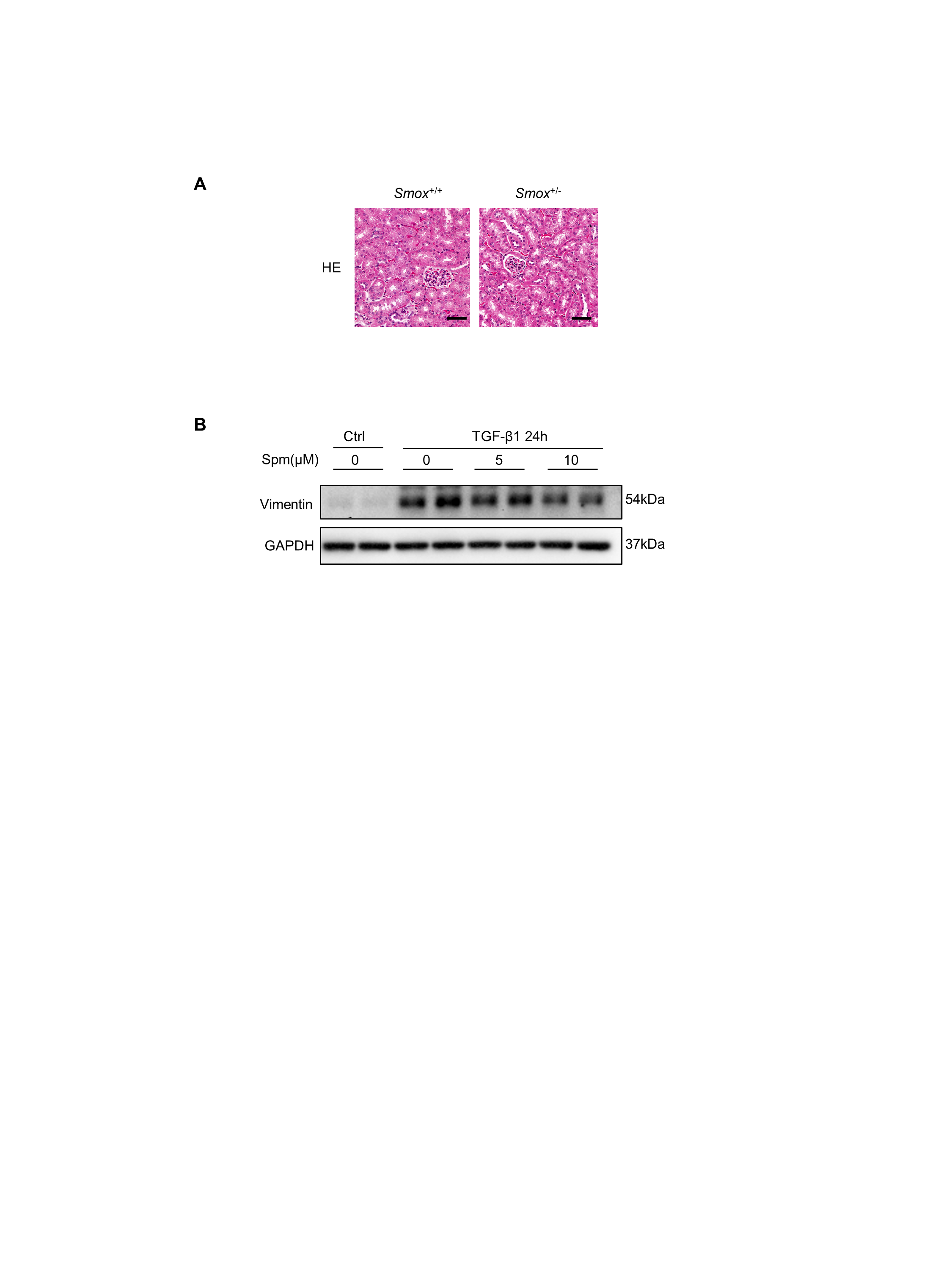


**Figure S4**  **A**) Representative images of kidney tissues under physiological conditions from *Smox*^+/+^ and *Smox*^+/-^ mice with HE staining. Scale bar, 50μm. **B**) mTECs were preincubated with 5 and 10 µM of spermine (Spm) for 12 hours and then treated with 10 ng/mL of TGF-β1 for 24 hours. Vimentin was detected. GAPDH is used as a control.


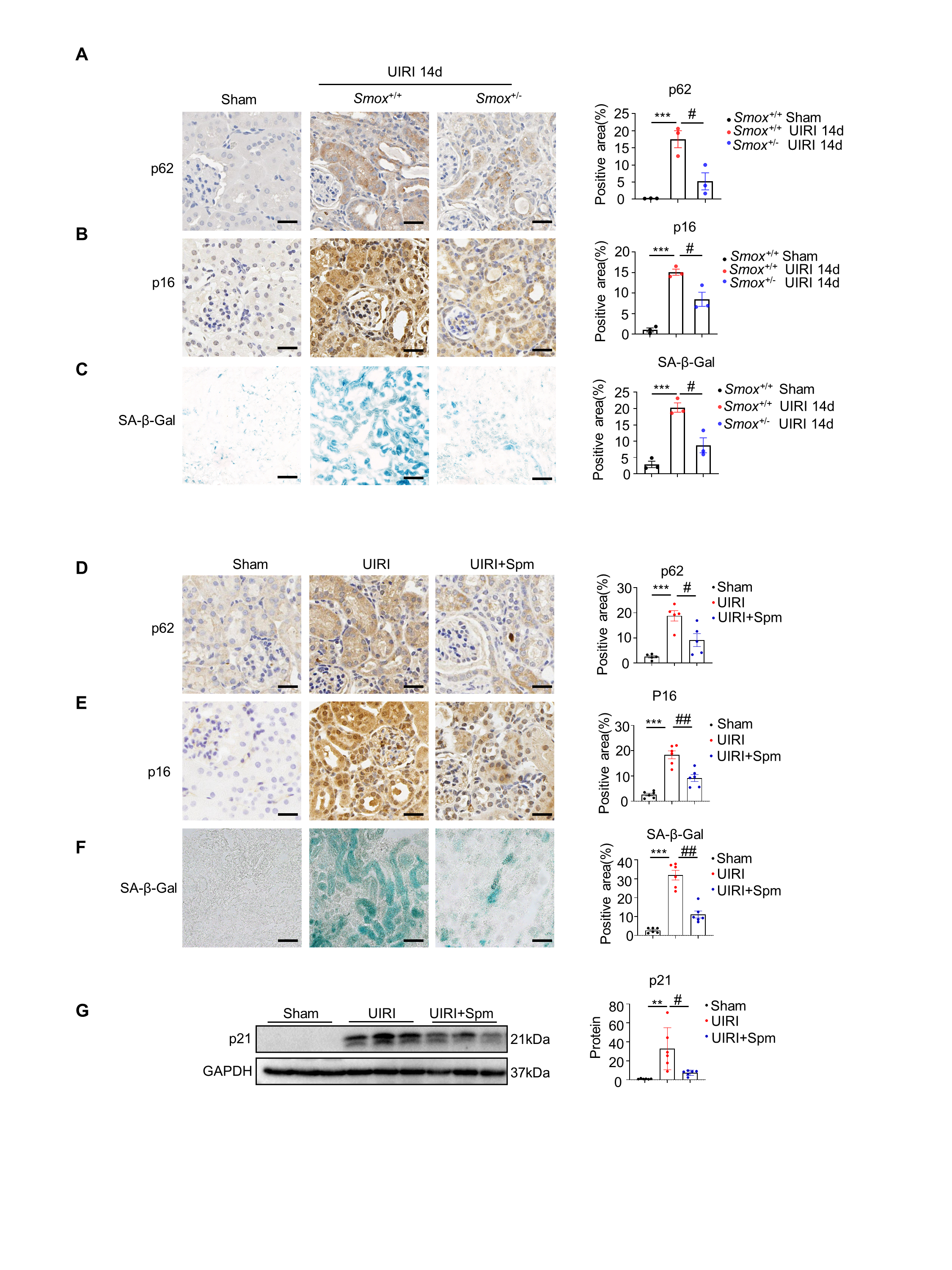


**Figure S5** Exogenous spermine or knockdown of SMOX inhibits cell senescence in the UIRI model. **A, B**) Representative micrographs and quantitative data with immunohistochemistry staining of p62 and p16 in the kidneys of *Smox*^+/+^ and *Smox*^+/−^ mice. Scale bar, 25 μm. ****P* < 0.001 versus the sham; #*P* < 0.05 versus *Smox*^+/+^ UIRI (n = 6). **C**) Representative micrographs and quantitative data with SA-β-gal activity staining in the kidneys of *Smox*^+/+^ and *Smox*^+/-^ mice. Scale bar, 50 μm. ****P* < 0.001 versus the sham; #*P* < 0.05 versus *Smox*^+/+^ UIRI (n = 6). **D, E**) Representative micrographs and quantitative data with immunohistochemistry staining of p62 and p16 are shown in different groups. Scale bar, 25 µm. ****P* < 0.001 versus the sham; #*P* < 0.05, ##*P* < 0.01 versus UIRI (n = 6). **F**) Representative micrographs and quantitative data with SA-β-gal activity staining are shown in different groups Scale bar, 50 µm. ****P* < 0.001 versus the sham; ##*P* < 0.01 versus UIRI (n = 6). **G**) Representative western blotting and quantitative data showing the expression of p21 in the kidneys of different groups. GAPDH was used as a control (n=6). Data are expressed as means ± SEM, ***P* < 0.01 versus sham controls; #*P* < 0.05 versus UIRI with vehicle (n = 6).


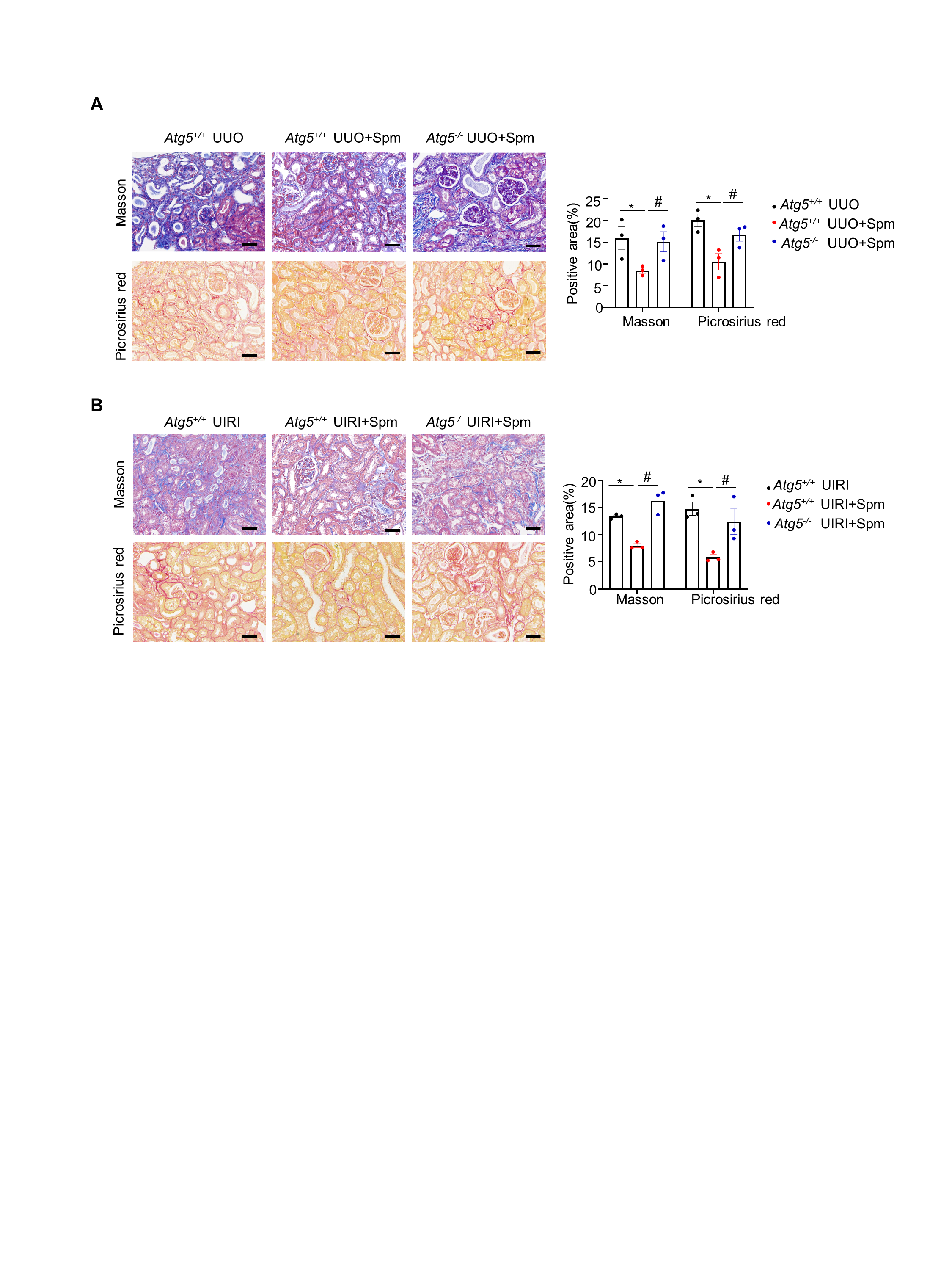


**Figure S6** Exogenous spermine alleviates renal fibrosis in CKD models of *Atg5*-deficient mice. Representative images of kidney tissues from indicated groups with either Masson trichrome staining (upper panel) or Picrosirius red staining (lower panel). Scale bar, 50 µm. Data are expressed as means ± SEM. **A**) **P* < 0.05 versus *Atg5*^+/+^ UUO; #*P* < 0.05 versus *Atg5*^+/+^ UUO with spermine supplementation (n = 3). **B**) **P* < 0.05 versus *Atg5*^+/+^ UIRI; #*P* < 0.05 versus *Atg5*^+/+^ UIRI with spermine supplementation (n = 3).


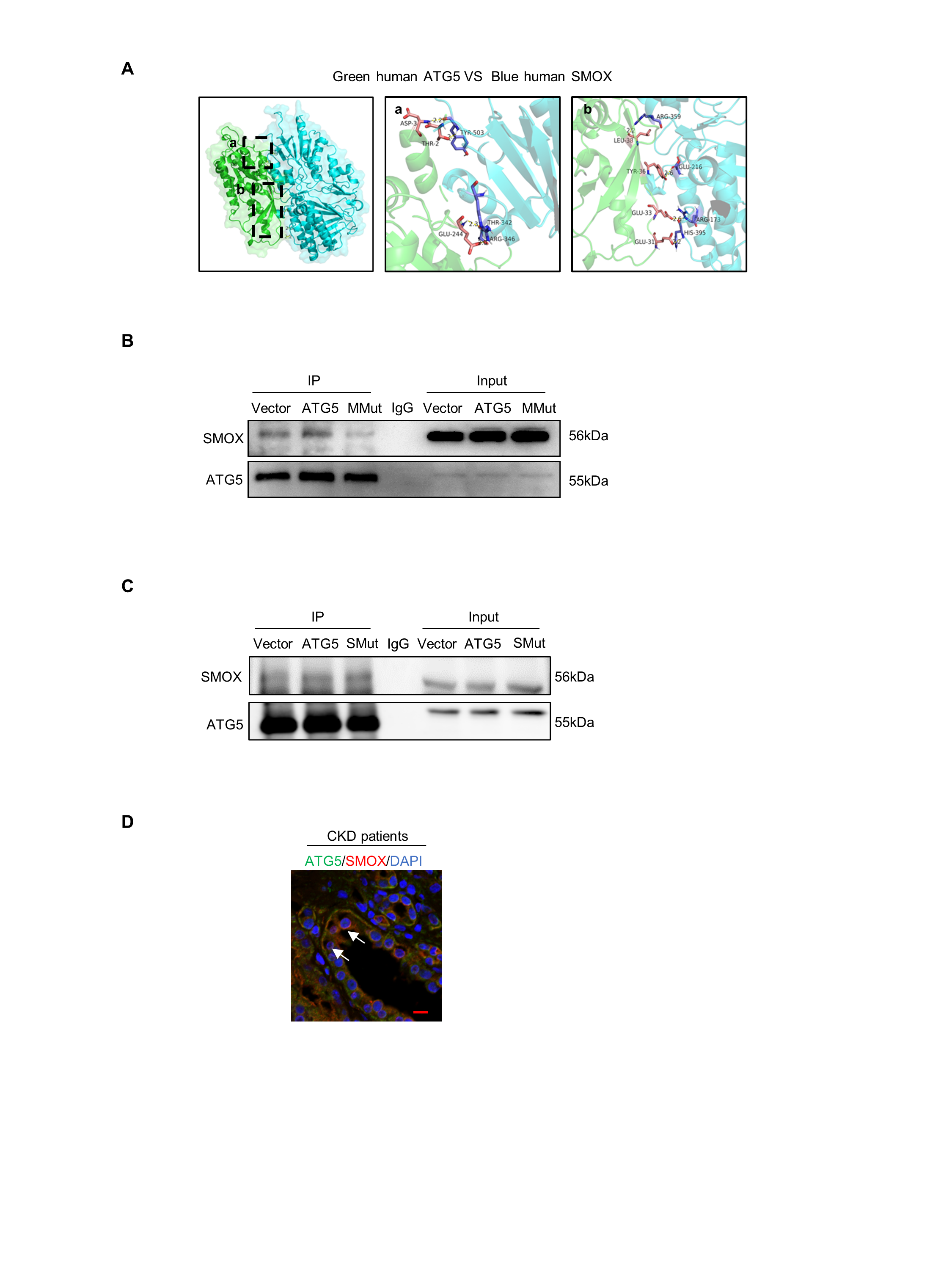


**Figure S7** ATG5 interacts with SMOX. **A**) Images represent the three-dimensional structure of human ATG5 (green) and SMOX (blue) proteins, and the dotted box indicates the binding site of ATG5 and SMOX proteins. **B**) mTECs were transfected with wild-type ATG5 (ATG5) or ATG5 multiple-site-mutated (MMut) plasmid. Co-immunoprecipitation was used to detect the binding of ATG5 to SMOX. **C**) mTECs were transfected with wild-type ATG5 (ATG5) or ATG5 E244A-mutated (SMut) plasmid. Co-immunoprecipitation was used to detect the binding of these ATG5 to SMOX. **D**) Double immunofluorescence staining of ATG5 and SMOX on kidney sections in CKD patients. Scale bar, 10 µm.

**Experimental Section**

*Cell proliferation assay* A BeyoClick™ EdU-488 imaging kit (Cat# C0071S, Beyotime Biotechnology, Shanghai, China) was used to assess cell proliferation according to the manufacturer’s instructions. Briefly, cells were seeded in a 12-well plate and treated with TGF-β1 (5 ng mL^-1^) in the absence or presence of spermine (10 × 10^−6^ M) for 24 hours, followed by incubation with 0.5mL EdU for 4 hours at 37°C. After washing with PBS three times, the cells were fixed with 4% paraformaldehyde and permeabilized with 0.3% Trixon-100. Cells were counterstained with Hoechst 33342. Cell proliferation was assessed either by flow cytometry (Beckman, USA) or imaged with a confocal microscope (LSM710, Carl Zeiss, Germany).

*SA-β-gal assay* Cells or frozen sections were performed SA-β gal activity using the Senescence β-Galactosidase Staining Kit (Cat#C0602, Beyotime), according to the manufacturer’s instructions. In brief, samples were fixed with 4% paraformaldehyde at room temperature for 15 minutes and immersed with freshly prepared staining solution overnight at 37℃. Images were captured under a microscope (Olympus BX51, Center Valley, PA).

*Quantitative real-time PCR (qRT-PCR)* Total RNA was extracted from either the kidneys or the cells the RNAiso Plus reagent (Cat#9108, Takara Biotechnology, Dalian, China) followed by cDNA synthesis using HiScriptII QRT SuperMix for qPCR (Cat#R223-01, Vazyme, Nanjing, China). Quantitative real-time PCR was performed by using SYBR Green PCR Master Mix (Cat#Q131-02, Vazyme) on a LightCycler 96 Instrument (Roche, Mannheim, Germany). Primers for genes of interest generated, and the oligonucleotide sequences are shown in Table S4.

*Co-Immunoprecipitation assays and Western blotting analysis* Briefly, cell lysates were prepared and immunoprecipitated with indicated antibodies. Immunocompleces were gathered using protein A/G Magnetic Beads (Cat#B23201, Bimake, Shanghai, China), suspended in 50 µl 1 × SDS buffer (Cat#P0015, Beyotime), and then loaded on SDS-PAGE.

Cells or kidney tissues were lysated, and protein expression was analyzed by western blotting analysis as previously described^[38]^. The primary antibodies used were as follows: Fibronectin (1:1000, Cat#2413, Abcam, Cambridge, MA, USA); Collagen I (1:1000, Cat#BA0325, Boster Pleasanton, CA, USA); α-SMA (1:1000, Cat#A5228, Sigma); LC3A/B (1:1000, Cat# 12741, CST, MA, USA); Atg5 (1:1000, Cat#108327, Abcam); P62 (1:1000, Cat#PM045, MBL); P53 (1:1000, Cat#2524, CST); P21 (1:1000, Cat#109199, Abcam); GAPDH (1:2000, Cat#2218, CST); β-Actin (1:2000, Cat#3700, CST); Vimentin (1:2000, Cat#92547, Abcam); SMOX (1:1000, Cat#15052-1-AP, Proteintech, Wuhan, China); P21(1:1000, Cat#109199, Abcam); the secondary antibodies were peroxidase-conjugated goat anti-rabbit (1:5000, Cat#A0208, Beyotime) or anti-mouse (1:5000, Cat#A0216, Beyotime). Densitometry analysis was performed using the Image J software (Wayne Rasband National Institutes of Health, USA).

*Histology, immunohistochemical and* *immunofluorescence staining* Paraffin embedded kidney sections were performed with Sirius red (Cat#ab150681, Abcam) and Masson trichrome staining (Cat#g1006, Servicebio, Wuhan, China) according to the manufacturer’s instructions. Immunohistochemical and immunofluorescence staining were conducted as previously described^[48]^. The primary antibodies used were as follows: SMOX (1:100, Cat#15052-1-AP, Proteintech); SAT1 (1:100, Cat# 10708-1-AP, Proteintech); α-SMA (1:100, Cat#A5228, Sigma); P62 (1:100, Cat# PM045, MBL, Japan); Vimentin (1:100, Cat#92547, Abcam); E-Cadherin (1:50, Cat#610181, BD bioscience, USA); P16 (1:200, Cat# ab189034, Abcam); For IF staining, we used the following secondary antibodies; Alexa Fluor 488 goat anti-rabbit IgG (Cat# A0423, Beyotime) and Alexa Fluor 555 donkey anti-mouse IgG (Cat# A0460, Beyotime).

*Proteomic analysis* Kidney tissue from *Smox^+/-^* and *Smox^+/+^* mice at 10 days after UUO were lysated. Protein extracts were precipitated with cold acetone, followed by digestion with trypsin. For digestion, the protein solution was reduced with 5 mM dithiothreitol for 30 min at 56 °C and alkylated with 11 mM iodoacetamide for 15 min at room temperature in darkness. The protein sample was then diluted by adding 100 mM TEAB to urea concentration less than 2 M. Finally, trypsin was added at 1:50 trypsin-to-protein mass ratio for the first digestion overnight and 1:100 trypsin-to-protein mass ratio for a second 4 h-digestion. Finally, the peptides were desalted by C18 SPE column. The peptides were lyophilized and redissolved in water with 20 µL 0.1% formic acid. The supernatant was directly loaded onto analytical column (NanoViper. 75um ID.x 25cm, packed with Acclaim PepMap100 C18, 2µm nano viper FS) and Nano Trap Column (NanoViper, 75um I.D.x 2 cm, packed with Acclaim PepMap100 C18, 3µm) of Ultra high-resolution Orbitrap exploris 480 Mass Spectrometer (Thermo Fisher Scientific, USA). Bioinformatics methods, including GO annotation and functional enrichment, were used to identify. The mass spectrometry proteomics data have been deposited to the ProteomeXchange Consortium (http://proteomecentral.proteomexchange.org) via the iProX partner repository with the dataset identifier PXD045175.

*Molecular docking of proteins* The 3D molecular structure of ATG5 (Human Q9H1Y0, Mouse Q99J83) and SMOX (Human Q9NWM0, Mouse Q99K82) was obtained from the PDB database (http://www.rcsb.org/). Proteins were retreated by removing water, adding hydrogens and charges, and extracting ligand structure by Discovery studio software (Dassault Systèmes, USA). The molecular docking was performed by the Zdock module with an angular step size of 6^0^. Protein structures were visualized and analyzed by the popular molecular graphics system PyMOL (Schrödinger, USA).

AlphaFold 2.3.0 represents a version of DeepMind's protein structure prediction tool that includes capabilities for predicting the structures of multimers, or protein complexes. The protein structure of SMOX ( MOUSE Q99K82 ) and ATG5 (Mouse Q99J83)、ATG7 ( Mouse Q9D906) and ATG3 (Mouse Q9CPX6) were used to assess the probability of contact and distance predictions between different protein chains. The DockQ value of protein-protein complexes needs to be more than 0.23 to get acceptable quality.
